# Exploring Diagnostic Priorities: The Role of Colonic Manometry in Evaluating Pediatric Patients with Intractable Idiopathic Constipation Prior to Sacral Nerve Stimulation

**DOI:** 10.3390/children11070768

**Published:** 2024-06-25

**Authors:** Lev Dorfman, Khalil El-Chammas, Azadvir Singh, Lin Fei, Sherief Mansi, Neha R. Santucci, Ajay Kaul

**Affiliations:** 1 Division of Pediatric Gastroenterology, Hepatology and Nutrition, Cincinnati Children’s Hospital Medical Center, Cincinnati, OH 45229, USA; lev.dorfman@cchmc.org (L.D.);; 2 Department of Pediatrics, University of Cincinnati College of Medicine, Cincinnati, OH 45229, USA; 3 Biostatistics, Cincinnati Children’s Hospital Medical Center, Cincinnati, OH 45229, USA

**Keywords:** colonic manometry, sacral nerve stimulation, constipation, refractory constipation, pediatric, motility

## Abstract

Background: Despite the limited understanding of its precise mechanism of action, sacral nerve stimulation (SNS) has proven to be helpful for pediatric patients with constipation, particularly those with fecal incontinence. It is unclear whether the outcome of SNS is impacted by normal or abnormal colonic motility. Our study aimed to determine whether colonic manometry results had an impact on the outcome of SNS as a treatment in pediatric patients with refractory idiopathic constipation. Methods: Electronic medical records of patients with idiopathic constipation who underwent colonic manometry and SNS placement at our center over 6 years were reviewed. A comparison of post-SNS outcomes was performed between patients with normal and abnormal colonic manometry studies. Results: Twenty patients [12 (60%) females, median age of 10.2 years] met inclusion criteria, with fecal incontinence in 12 (60%) and abnormal colonic manometry in 6 (30%). Significantly more patients had an improvement in fecal incontinence following SNS placement (*p* = 0.045). There were no significant differences in post-SNS constipation outcome measures between patients with normal versus abnormal colonic manometry. Conclusions: Colonic manometry did not help with patient selection for those being considered for SNS therapy. Our findings do not support performing colonic manometry as a screening prior to SNS placement.

## 1. Introduction

Constipation is highly prevalent in children, with an estimated global prevalence of almost 10% [1]. The majority of pediatric patients with constipation can be effectively managed through dietary adjustments and behavioral training [1]. More severe cases are treated with oral laxatives and/or rectal therapy. While most patients do not need diagnostic evaluation, those with refractory constipation (symptoms that are refractory to medical treatment) may benefit from further assessment [2]. There are several studies that can evaluate the various aspects of the dynamics of defecation, such as colonic transit, the structure/anatomy of the colon and anorectal regions, their innervation, and neuromuscular integrity [1,3]. These tests include transit marker study, contrast enema/defecography, MRI of the lumbosacral spine, electromyography, and anorectal and colonic manometry [4]. Anorectal manometry is a short study that obtains objective metrics of the internal and external anal sphincters. This study assesses several different aspects of the rectum and the anus: neuromuscular integrity, sensation, baseline pressures, reflexes, and muscle coordination [5].

Colonic manometry is a pressure catheter-based diagnostic procedure that assesses colonic motor activity by measuring the intraluminal pressure of the colon and allows visualization of the colon contraction patterns [6]. It is able to distinguish neuropathic from myopathic motility disorders. Due to its complexity necessitating colonic catheter placement under general anesthesia with the use of fluoroscopy, as well as performing the study in an inpatient setting, colonic manometry is reserved for the evaluation of patients with refractory constipation, persistent fecal incontinence, post-surgical patients with Hirschsprung’s disease with persistent defecatory problems, and pediatric intestinal pseudo-obstruction [7]. Colonic manometry was found to be beneficial in assessing the need for surgical intervention in pediatric patients with constipation and prior to the reconnection of patients with diverting ostomies [8,9]. The European Society for Paediatric Gastroenterology, Hepatology, and Nutrition (ESPGHAN) and North American Society for Pediatric Gastroenterology, Hepatology, and Nutrition (NASPGHAN) guidelines on the management of pediatric functional constipation recommend that patients with refractory constipation should be assessed by colonic manometry and treated with surgical therapies, anal sphincter botulinum toxin injection, or sacral nerve stimulation (SNS) [2].

Stimulation of the sacral nerves was initially reported to be an effective adjunctive treatment for urinary incontinence, and it was later introduced as a treatment modality for pediatric patients with refractory constipation and fecal incontinence with mixed results [10,11,12,13]. The stimulation is performed via an electrode, which is surgically placed through the third sacral foramen. SNS placement is performed as a two-stage procedure, with initial temporary placement for approximately 2 weeks for evaluation of response, followed by permanent placement in a subcutaneous pocket in patients who have demonstrated a beneficial response [14]. Adverse events and complications range from minor local pain and neurological symptoms to local wound infections and the need for repeated surgical intervention, but the majority of parents report positive health-related benefits [15,16]. No risk factors have been identified that predict complications after SNS placement except for more lead migration, for unexplained reasons, in children having SNS placement for bladder symptoms [17].

Although the precise mechanism of its beneficial action in patients with constipation is unclear, several studies assessing the effectiveness of SNS in some pediatric patients with constipation have shown promising results, especially in those with fecal incontinence [15,18]. In addition, SNS was shown to reduce the frequency of abdominal symptoms and increase continence in a mixed pediatric cohort who underwent SNS placement for constipation with fecal incontinence following a bowel management program [16]. When compared to antegrade continence enemas (ACE), SNS was found to be more beneficial in improving fecal incontinence in pediatric patients and reducing antegrade continence enema use in patients who received ACE prior to SNS placement [18,19]. The optimal timing of SNS placement in patients with refractory constipation is not known, and currently, SNS placement is suggested in patients with medically refractory constipation prior to other surgical interventions such as diversion and segmental colonic resection [2,3]. Interestingly, SNS is suggested by ESPGHAN/NASPGHAN to be an optional treatment in patients with normal as well as abnormal colonic manometry findings [2]. Nevertheless, the role of colonic manometry in identifying colonic motility disorder in the selection of patients for SNS has not been previously assessed, and it is unclear whether the outcome of SNS is impacted by normal or abnormal colonic motility.

The aim of our study was to determine if the results of colonic manometry had any impact on the outcome of SNS placement as an adjunctive treatment in pediatric patients with medically refractory idiopathic constipation.

## 2. Materials and Methods

Electronic medical records of all patients who underwent sacral nerve stimulation placement in our center over a period of 6 years were reviewed.

Inclusion criteria: (1) Patients with medically refractory idiopathic constipation; (2) Younger than 21 years; (3) Underwent evaluation with a colonic manometry study at the Neuro-Gastroenterology and Motility Disorders Center at Cincinnati Children’s Hospital Medical Center (CCHMC) prior to SNS placement. Patients who did not complete colonic manometry were excluded.

Data reviewed included demographics, co-morbidities, symptoms, medications, results of a water-soluble contrast enema interpreted by a neurogastroentrologist, indication for colonic manometry, colonic manometry findings and tracings, and its interpretation by a neurogastroenterologist, surgical history, anorectal manometry findings, SNS-associated complications, SNS removal, and outcomes (bowel movement frequency, bowel movement consistency, urinary incontinence, fecal soiling, change in laxative regimen). All patients were continued on the laxative and/or rectal therapies that they were on, and the regimens were adjusted after SNS placement based on the clinical response pursuant to the provider’s decision. Images of water-soluble contrast enemas were independently analyzed by two experienced pediatric neurogastroenterologists and categorized as normal, mega rectum, mega colon, and redundant sigmoid colon. Improvement in fecal soiling and urinary incontinence was defined as a reduction of weekly events by at least 50%. An increase in bowel movements was defined as a weekly increase of at least 50% in the frequency of bowel movements. Improvement or worsening in bowel movement consistency was defined as a descriptive change from hard to soft stool or vice versa. De-escalation in laxative use was determined by a reduction in the number of regimens used, cessation of enema usage, or a reduction in the frequency of antegrade enema flushes by at least 50%.

### 2.1. Colonic Manometry

Colonic manometry studies were performed according to published consensus guidelines using Laborie (Medical Measurement System, Enschede, The Netherlands) systems [5]. The placement of manometry catheters was performed endoscopically under general anesthesia by a neurogastroenterologist at our center after an inpatient bowel clean-out with nasogastric administration of GoLytely. To mitigate the impact of anesthesia on colonic motility, all studies were conducted the next day following the endoscopic placement [20].

**Figure 1:**
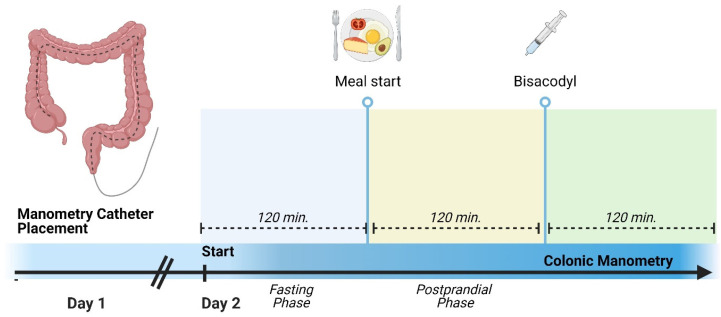
Colonic manometry protocol. Created with BioRender.com.

Water-perfused catheters (8 to 16 channels with 3–5 cm spaced sensors) or solid-state catheters or solid-state catheters (36 channel with 2 cm spaced sensors) were used depending on age, length of the colon, and the provider’s preference. The studies were conducted following radiographic verification of catheter location and lasted at least 6 h, including at least 2 h of fasting, followed by a meal with a minimal caloric intake of at least 400 kcal or 20 kcal/kg [5,21]. The post-prandial recording lasted 1.5–2 h. Colonic stimulation with bisacodyl (0.2 mg/kg up to a maximal dose of 10 mg) administration in the right colon/cecum was provided through the central channel of the colonic manometry catheter, as needed based on the provider’s decision during the study and if no high-amplitude propagating contractions were noted during the fasting and feeding phases [22]. A second dose of bisacodyl was administered if at least one high-amplitude propagation contraction was not noted in all colonic segments [23]. The post-stimulation phase lasted up to 120 min (see colonic manometry protocol in Figure 1). The interpretation of colonic manometry studies was performed by a neurogastroenterologist in our center. High amplitude propagating contractions were defined as contractions propagating over at least 30 cm of the length of the colon with an amplitude of at least 65 mmHg [6]. The presence of a gastrocolonic response was defined as an increase in the post-prandial motility index by at least 15% from baseline. The motility index was calculated by manometry software per previously published standards [24]. The colonic manometry studies were interpreted as normal if all three of the following criteria were fulfilled: 1. Presence of high-amplitude propagating contractions throughout the colon during at least one phase of the study (fasting, post-prandial, or post-stimulation with bisacodyl); 2. The presence of a gastrocolonic response; 3. Absence of retrograde or simultaneous segmental contractions.

### 2.2. Statistical Analysis

Patients were divided into two groups: those with normal colon manometry and those with abnormal colon manometry. Demographic characteristics were summarized and compared between the normal colonic manometry and abnormal colonic manometry groups. Continuous variables are presented as means ± standard deviations for normally distributed variables and as medians with interquartile ranges (IQRs) for non-normally distributed variables. Comparisons were made either with a two-sample *t*-test or the Wilcoxon test. Qualitative or categorical variables are expressed as numbers and proportions and were compared with Fisher’s exact test. The comparison between normal results of the colonic manometry test was conducted using Fisher’s exact test. Changes in bowel movements, such as those before and after SNS, were evaluated with a McNemar’s test, further confirmed by a likelihood ratio test. The study protocol was approved by the institutional review board (approval number 2022-0568).

## 3. Results

Twenty pediatric patients with intractable idiopathic constipation and adjunctive SNS placement met our inclusion criteria. Of these 20 patients, 12 (60%) were female. The median age of the 20 patients was 10.2 years (IQR: 7.5, 14.4, range: 5.5–20.5 years). The median follow-up duration after SNS placement was 20.5 months (range 7–42 months), with 85% (17/20) following for at least 12 months. Fecal incontinence was noted in 12 (60%) of patients and urinary incontinence in 7 (35%). The characteristics of the cohort are presented in Table 1.

The water-soluble contrast enema study was normal in 9 (45%) patients and showed megarectum in 7 (35%) patients. Redundant sigmoid colon was noted in 6 (30%) patients, and megacolon in 1 (5%) patient. In 3 out of 6 patients (50%) with prior cecostomy, dilation of the cecum/ascending colon was noted on water-soluble contrast enema. A majority (*n* = 12; 60%) of the patients were treated by a combination of oral and rectal laxatives, while 4 (20%) patients were exclusively on daily rectal therapy and 4 (20%) patients were exclusively on oral laxatives.

### 3.1. Colonic Manometry

**Figure 2:**
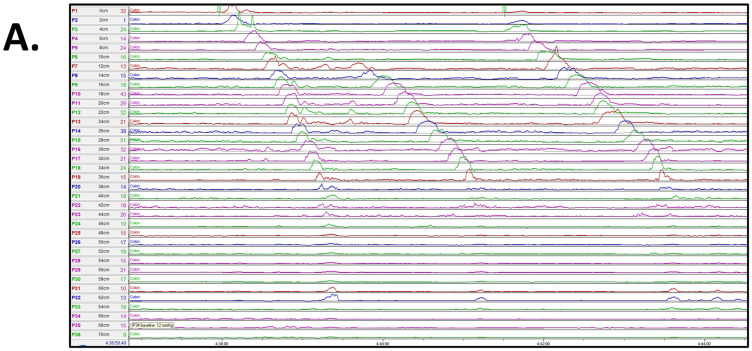

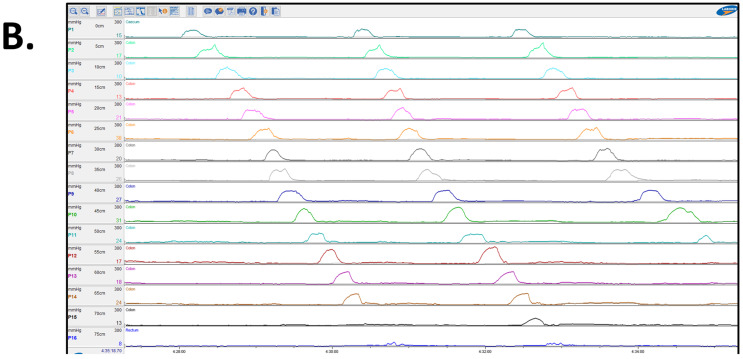
(**A**) Abnormal colonic manometry in an 11-year-old male, showing termination of high amplitude propagating contractions in the distal (left) colon. (**B**) Normal colonic manometry in a 10-year-old female with high amplitude propagating contractions throughout the colon.

Colonic manometry was abnormal in 6 (30%) patients with a lack of high-amplitude propagating contractions in the distal (left) colon, despite stimulation with intracolonic administration of bisacodyl (Figure 2). All six of these patients were reported to have a dilated rectosigmoid on a water-soluble contrast enema. The remainder of the patients, 14 (70%), had normal colonic manometry. Improvement in the frequency of bowel movements, fecal incontinence, and reduction of laxative use after SNS placement in patients with abnormal colonic manometry were noted in 4/6 (66%) patients (see below).

### 3.2. Cecostomy and Sigmoidectomy

Six (30%) patients underwent SNS placement after cecostomy, while 2 (10%) patients had cecostomy placement (for antegrade enemas—ACE) after SNS placement. Six patients (30%) had sigmoidectomy (2 of them post-SNS placement). The change in constipation parameters based on cecostomy status is provided in Table S1 in the Supplementary Data.

### 3.3. Frequency of Bowel Movements

Following SNS placement, the number of bowel movements increased in 4 (20%) patients, with 14 (70%) of patients reporting no change in bowel movement frequency and 2 (10%) reporting worsening with a reduction in bowel movement frequency. All patients met the Rome 4 criteria for functional constipation, and the average bowel movements per week prior to SNS placement was 7.9 per week vs. an average of 7.6 bowel movements per week after SNS placement (*p* = 0.76). Among patients with abnormal colonic manometry (6, 30%), one patient (16.7%) had an increase in bowel movements, while five patients (83.3%) had no change in bowel movements, with no statistically significant difference when compared to patients with normal colonic manometry.

### 3.4. Bowel Movement Consistency

Stool consistency improved in 2 (10%) patients, with no change in stool consistency in 18 (90%) patients following SNS placement. Colonic manometry was normal in all patients who experienced improvement in stool consistency.

### 3.5. Fecal Soiling

Fecal incontinence resolved in 3 out of 12 (25%) patients and decreased in 5 (41.7%) patients. No change in the frequency of fecal soiling events was noted in three (25%) patients, and one patient (8.3%) had worsening fecal incontinence. The median reduction in fecal soiling events in the entire cohort was from 8.2 to 3.8 events per week. Statistically significant improvement was noted among patients with fecal soiling after SNS placement (*p* = 0.045, see Table 2). In the six patients with abnormal colonic manometry (all with dilation of the rectosigmoid colon), improvement in incontinence episodes was noted in four (66.7%) patients, with no change in fecal incontinence episodes in two (33.3%) patients. No difference in fecal incontinence was noted in patients with normal or abnormal colonic manometry (*p* = 0.4) after SNS placement.

### 3.6. Change in Laxative Regimen

**Figure 3:**
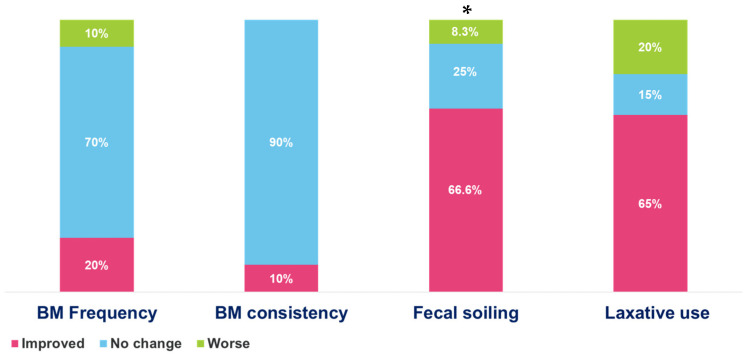
Effect of SNS on constipation in pediatric children with idiopathic constipation. *—statistically significant difference (*p* < 0.05).

A reduction in laxatives and enemas use was noted in 13 (65%) patients, no change in 3 (15%) patients, and an increase in laxative use in 4 (20%) patients, *p* = 0.052. Among patients with abnormal colonic manometry (*n* = 6), a reduction in laxative regimen was reported in 3 (50%), no change in 2 (33%), and an increase in laxative regimen was reported in 1 (16.6%). The effect of SNS on bowel movements and fecal soiling is presented in Figure 3.

In patients with SNS placement after cecostomy (*n* = 6), improvement was noted in 3 (50%)—one (16.6%) patient had an increase in bowel movements, and 2 (33.3%) had improvement in soiling. Comparing patients with prior cecostomy to patients without cecostomy, no significant change was noted between the groups in bowel movement frequency, consistency, fecal soiling events, or reduction in laxatives (see Supplementary Table S1).

Sigmoid resection prior to SNS placement (*n* = 4) or after SNS placement (*n* = 2) did not have any impact on any of the outcome measures studied (frequency, consistency, soiling, or change in laxative regimen). Colonic manometry findings were abnormal in 50% of patients who later underwent sigmoid resection and SNS placement.

### 3.7. Urinary Incontinence

In patients with urinary incontinence (*n* = 7), improvement in urinary incontinence events was noted in 5 (71.4%) patients, with resolution of urinary incontinence in two (28.6%) of them. No change in urinary incontinence was noted in 2 (28.6%) patients, but there was an improvement in fecal soiling and a decrease in laxative use in both. Overall, fecal soiling was noted among 6 out of 7 patients with urinary incontinence, and improvement in fecal soiling post-SNS placement was noted in 4 (66%) (see Supplementary Table S2 for data regarding constipation parameters in patients with urinary incontinence).

### 3.8. Normal vs. Abnormal Colonic Manometry

Out of 6 patients who had an abnormal distal (left) colonic manometry, improvement in constipation parameters after SNS placement was noted in 4 (66%). The response to SNS in patients with abnormal colonic manometry is presented in Table 3. Comparing patients with normal colonic manometry with those with abnormal colonic manometry, no statistically significant differences were noted in age, sex, constipation outcome measures (bowel movement frequency, bowel movement consistency, fecal soiling events, and change in laxative regimen) or post-SNS laxative change, as well as in other factors studied, including the presence of cecostomy and sigmoidectomy (see Table 4).

### 3.9. SMS Removal/Replacement

The need for re-operation was noted in 6 (30%) patients, with 2 (10%) patients who had SNS removal due to failure to respond, 1 (15%) patient needed surgery due to an exposed battery, and 3 (15%) patients who needed SNS lead repositioning.

## 4. Discussion

This is the first pediatric study assessing the role of colonic manometry prior to SNS placement and outcome in pediatric patients with medically refractory idiopathic constipation.

In our cohort, the only statistically significant improvement after adjunctive SNS therapy was a reduction in episodes of fecal incontinence and a trend toward reduction in laxative and rectal therapy. There was no noteworthy change in bowel movement frequency or consistency, with a trend toward a reduction in laxative use. This apparently suggests that SNS improves the evacuation of stool load (volume) from the left colon without impacting the frequency or consistency of stools. This most likely occurs as a result of the SNS stimulating primarily the anal sphincters and distal colon, as they are supplied by the sacral nerves. It is plausible that SNS improves the baseline tone and function of the anal sphincters, including the colo-anal reflex, which occurs in anticipation of stool migrating down towards the rectum [25,26,27]. We propose that the effect of SNS is two-fold. Firstly, it likely generates a stronger propagated contraction in the left colon, including in those that may not have had that prior to SNS placement, as noted on pre-placement colonic manometry. Secondly, it may induce the anal sphincters to work more efficiently by virtue of timely and adequate contraction and relaxation of the anal sphincters. Together, these two factors may facilitate a more complete evacuation of stool from the rectal reservoir and thereby decrease fecal incontinence without impacting the frequency or consistency of stool.

Defecation occurs as a result of high amplitude propagating contractions that start in the proximal colon and migrate distally, moving fecal contents aborally, referred to as mass movement in contrast studies. Not all high-amplitude propagating contractions result in a bowel movement. In a pediatric case series from our center, turning the SNS on during a colonic manometry study was shown to induce propagating contractions not only in the distal but also in the proximal colon in 50% of the patients [28]. Since SNS did not have a significant effect on the frequency of bowel movements in this study, it may imply that an increase in the colonic motor function caused by SNS may not be the main mechanism of action by which SNS improves outcomes in pediatric patients with refractory constipation.

The physiologic basis for improvement in urinary incontinence after SNS placement is probably similar to the improvement in bowel function, which is not exclusively induced by the effect of electric pulses on the bladder and the bowel but also by improvement in the central processing and reflex regulation induced by the stimulation of the sacral nerves. This mechanism is supported by previous studies in both animals and humans [29].

SNS was previously shown to be effective in patients with spinal malformations [30]. If the effect was limited to just the anal sphincters, it would indicate that in disorders of the anal sphincters, either congenital or acquired, SNS would not be effective in decreasing fecal incontinence. However, in a systematic review by Dewberry et al., the authors concluded that SNS cannot be definitively encouraged or discouraged in patients with anorectal malformations [31]. Improvement in defecatory problems reported in some cases with anorectal malformation that do respond to SNS is aligned with our finding in a pediatric case, demonstrating an increase in colonic motor activity after SNS is turned on during colonic manometry [28].

The outcomes of patients after SNS in our study are similar to previously described findings in pediatrics by Lu et al., who showed an improvement in fecal incontinence without significant changes in bowel movement frequency in a more heterogeneous group of pediatric patients [15]. Comparable outcomes were also noted in a 19-patient series of pediatric patients with idiopathic constipation treated with SNS that showed a decrease in fecal incontinence in 40% of patients at 6 months (compared to the improvement of fecal incontinence in 41% in our cohort with resolution of fecal incontinence of an additional 25%) [18]. However, the increase in frequency of bowel movements in our cohort was higher (40% vs. 21%) compared to the previously reported cohort by Vriesman et al.

Our findings are also aligned with the conclusion of a review of the effect of SNS on fecal incontinence and bowel movements by the Cochrane Library in adults with variable defecatory disorders [15,26]. Our study focused specifically on pediatric patients with idiopathic constipation and showed significant improvement in fecal soiling and a trend toward a reduction in the need for laxatives after SNS placement. We may need to study a larger cohort to confirm this finding.

### Role of Colonic Manometry Prior to SNS Placement

In our cohort, colonic manometry findings (normal or abnormal) did not impact the outcome of patients who had SNS placement. This goes along with published pediatric society guidelines (ESPGHAN and NASPGHAN) suggesting the potential use of SNS irrespective of the findings of colonic manometry [2]. Improvement in different constipation outcome measures was noted among patients in both groups, including improvement in fecal soiling in 4 out of 6 (66%) patients with abnormal colonic manometry. This implies that abnormal colonic manometry does not necessarily preclude SNS placement trials, as the improvement in symptoms in this cohort may be due to improvements in the motor function of the left colon and anal sphincters, as well as the induction of high-amplitude propagating contractions in the proximal colon that occur aborally.

Currently, colonic manometry and SNS placement are suggested almost as a last resort in the evaluation and treatment of pediatric patients with refractory constipation [2,6,32]. While colonic manometry has a role in guiding surgical interventions in patients with refractory constipation, we are still unclear on which would be the ideal group of patients who would benefit from it. Furthermore, the role of colonic manometry in the assessment of candidates for SNS remains unclear [8,9,33]. Our findings do not support an obligatory need for a normal colonic manometry study as an antecedent step prior to the SNS trial. As the performance of colonic manometry studies and placement of SNS are currently limited to tertiary referral centers worldwide, there is an urgent need for evidence-based guidelines and indications for evaluating pediatric patients with refractory constipation and when to contemplate SNS placement. A recent study reported that only 5.3% of pediatric patients had colonic manometry studies performed prior to SNS placement in a tertiary center that performs colonic manometry studies [18]. While this low number of colonic manometries performed in patients with SNS may reflect the local practice, further studies and data are needed to establish guidelines that will determine the evidence-based approach to patients with refractory idiopathic constipation.

It should be noted that all of our patients had high-amplitude propagating contractions in at least part of the colon, which indicates that the neuromuscular function was intact at least in that segment. It is not known if SNS would be effective in patients with colonic inertia when there is a complete lack of propagated contractions in the colon. Even though SNS did seem to help some patients in this subset with the segmental presence of high-amplitude propagating contractions, it is unclear if it did so by improving colonic motor function, by its action on the anal sphincters, or both. The effect of SNS on this group needs further evaluation in a larger cohort.

SNS was beneficial in patients with a combination of urinary and fecal incontinence, showing improvement in at least one of the conditions in all patients (see Supplementary Data). SNS was initially introduced as a therapy for patients with urinary incontinence and only later as a possible therapeutic option in cases of refractory constipation. A similar mechanism of action of SNS on bladder and bowel continence was previously described and reviewed [29]. Previously published studies and our current findings suggest that SNS as a therapeutic modality in patients with combined urinary and fecal incontinence should be considered [12,15,25,29].

The main limitation of our study is the relatively small number of patients and the retrospective design. It can be challenging to characterize constipation outcomes, especially bowel movement consistency and frequency, in a retrospective study. In addition, the laxative treatment was not standardized, and the variability in treatment could affect data interpretation. Although larger prospective studies of pediatric patients with abnormal colonic manometry prior to SNS placement are needed, this would be a formidable task as both colonic manometry and SNS are currently available at only a handful of pediatric centers worldwide.

## 5. Conclusions

To conclude, despite concomitant antegrade enemas via cecostomy in 8 (40%) and sigmoid resection in 6 (30%) patients, placement of SNS did not uniformly improve outcomes of idiopathic constipation in children with medically refractory idiopathic constipation. However, SNS was found to be specifically effective in improving fecal incontinence, with additional value in patients with a combination of fecal and urinary incontinence. Colonic manometry did not help with patient selection considered for SNS therapy, and our findings do not support performing colonic manometry as a mandatory screening prior to SNS placement. This is the first evidence that supports the European and North American Societies for Pediatric Gastroenterology, Hepatology, and Nutrition (ESPGHAN/NASPGHAN) position that SNS can be recommended in patients with refractory constipation without colonic manometry.

## Figures and Tables

**Table 1:** Characteristics of patients with idiopathic constipation and SNS placement.

Pediatric Patients with Idiopathic Constipation and SNS (*n* = 20)		
Age (median, IQR)		10.2 (7.5, 14.4)
Gender, female (*n*, %)		12 (60%)
Follow-up month, median (*n*, range)		20.5 (7–42)
Fecal incontinence (*n*, %)		12 (60%)
Urinary incontinence (*n*, %)		7 (35%)
Water-Soluble Contrast Enema	Normal	9 (45%)
	Megacolon	1 (5%)
	Megarectum	7 (35%)
	Redundant sigmoid	6 (30%)
Colonic Manometry, Abnormal (*n*, %)		6 (30%)
Laxatives (*n*, %)	Oral	4 (20%)
	Enemas/Flushes	4 (20%)
	Combined	12 (60%)

**Table 2:** Comparison of constipation parameters after SNS placement in pediatric children with idiopathic constipation.

	Improved	No Change	Worse	*p*-Value *
BM Frequency	4	14	2	0.683
BM consistency	2	18	0	0.48
Fecal soiling	8	11	1	0.045
Laxative change	13	3	4	0.052

BM—bowel movements. * McNemar’s test.

**Table 3:** The effect of SNS on patients with abnormal colonic manometry.

Patient	Urinary Incontinence	Fecal Incontinence	BM Frequency	BM Consistency	Laxative Use
1	Improved	Improved	No change	No change	No change
2	Not applicable *	Improved	Improved	No change	Decreased
3	No change	Improved	No change	No change	Decreased
4	Improved	No change	No change	No change	Increased
5	Not applicable *	No change	No change	No change	No Change
6	No change	Improved	No change	No change	Decreased

* Did not have urinary incontinence. BM—bowel movements.

**Table 4:** Comparison between patients with normal and abnormal colonic manometry.

		Normal Colonic Manometry (*n* = 14)	Abnormal Colonic Manometry (*n* = 6)	*p*-Value *
Sex, female *n*, (%)		10 (71.4%)	2 (33.3%)	0.16
Age, years (median (IQR))		13.5 (8.3–16.4)	7.5 (7.1–10.1)	0.06
Cecostomy, *n* (%)		5 (35.8%)	2 (33.3%)	0.64
Follow-up length, months (mean)		22.3	23.5	0.83
Rectosigmoid resection		3 (21.4%)	3 (50%)	0.3
Bowel movement frequency	Improved	3	1	1
	No change	9	5	
	Worse	2	0	
Bowel movement consistency	Improved	2	0	1
	No change	12	6	
	Worse	0	0	
Fecal soiling	Improved	4	4	0.4
	No change	9	2	
	Worse	1	0	
Laxative regimen	Reduce	10	3	0.3
	No change	1	2	
	Increase	3	1	

* Fisher’s exact test.

## Data Availability

The data presented in this study are available on request from the corresponding author due to privacy.

## Supplementary Materials

The following supporting information can be downloaded at: https://www.mdpi.com/article/10.3390/children11070768/s1, Table S1: Change in constipation parameters based on cecostomy status; Table S2: Constipation parameters among patients with urinary incontinence after SNS placement.
