# Supplementary material for: Exploring Diagnostic Priorities: The Role of Colonic Manometry in Evaluating Pediatric Patients with Intractable Idiopathic Constipation Prior to Sacral Nerve Stimulation
Source: Children (Basel). 2024 Jun 25;11(7):768. doi: 10.3390/children11070768 (PMC11274712; doi:10.3390/children11070768)
Supplement: Supplementary file 1 [file children-11-00768-s001.zip › children-3048809-supplementary.pdf]

Supplementary Data

Table S1. Change in constipation parameters based on cecostomy status.

		Improved	No Change	Worse	*p*-Value
BM Frequency	No cecostomy (n = 14)	3	9	2	1
	Prior cecostomy (n = 6)	1	5	0	
BM consistency	No cecostomy (n = 14)	0	14	0	0.079
	Prior cecostomy (n = 6)	2	4	0	
Fecal soiling	No cecostomy (n = 14)	6	7	1	1
	Prior cecostomy (n = 6)	2	4	0	
Laxative change	No cecostomy (n = 14)	10	1	3	0.303
	Prior cecostomy (n = 6)	3	2	1	

BM—Bowel movements.

Table S2. Constipation parameters among patients with urinary incontinence after SNS placement.

Patient	Urinary Incontinence	Fecal Incontinence	BM Frequency	BM Consistency	Laxative Use
1	Resolved	No change	No change	No change	Decreased
2	Resolved	Improved	Worsening	No change	Increased
3	Improved	Improved	No change	No change	No change
4	Improved	No change	No change	No change	Increased
5	Improved	No change	No change	Improved	Increased
6	No change	Improved	No change	No change	Decreased
7	No change	Improved	No change	No change	Decreased

BM—Bowel movements.
